# Genetic differentiation of geographic populations of *Rattus tanezumi* based on the mitochondrial *Cytb* gene

**DOI:** 10.1371/journal.pone.0248102

**Published:** 2021-03-18

**Authors:** Yingying Liu, Lisi Yao, Ying Ci, Xiaomei Cao, Minghui Zhao, Ying Li, XiaoLong Zhang

**Affiliations:** 1 Institute of Health Inspection and Quarantine, Chinese Academy of Inspection and Quarantine, Beijing, China; 2 Jiangxi International Travel Health Care Center, Nanchang, Jiangxi, China; National Cheng Kung University, TAIWAN

## Abstract

*Rattus tanezumi* is a common domestic rat and host of the bubonic plague pathogen in China and Southeast Asia (SEA). The origin, genetic differentiation and dispersal of *R*. *tanezumi* have received increasing attention from researchers. The population genetics of *R*. *tanezumi* based on its mitochondrial cytochrome b gene have been studied to explain the origin, relationships and dispersal of populations. In this study, we captured a total of 229 rats; morphological and molecular biological identification cytochrome oxidase subunit I (COI) confirmed 131 *R*. *tanezumi* individuals collected from 6 provincial areas, and their *Cytb* gene sequences were analyzed. The results showed that the population in Mohan (MH), Yunnan, had the highest genetic diversity, while that in Ningde (ND), Fujian, had the lowest. Tajima’s *D* statistic for all populations was negative and nonsignificant, indicating the possible expansion of *R*. *tanezumi* populations. Low gene flow occurred between the Zhangmu (ZM) *R*. *tanezumi* population and other populations, and the genetic differentiation among them was high. Furthermore, our analyses revealed the ZM lineage was the oldest lineage among the groups and diverged ~1.06 Mya, followed by the Luoyang (LY) lineages (~0.51 Mya) and Yunnan lineage (~0.33 Mya). In southeastern Yunnan, the Jinshuihe (JSH) and MH populations were more closely related to the populations in southeastern China (Fuzhou (FZ), ND, Quanzhou (QZ), Nanchang (NC)) and inland areas (Chongqing (CQ), LY) than to those in other areas of Yunnan (Jiegao (JG) and Qingshuihe (QSH)), indicating that *R*. *tanezumi* may have spread from southeastern Yunnan to the interior of China. In summary, *R*. *tanezumi* may have originated in ZM and adjacent areas, spread to Yunnan, and then spread from the southeast of Yunnan inland or directly eastward from ZM to inland China.

## Introduction

*Rattus tanezumi* belongs to Mammalia, Rodentia, Muridae, and *Rattus* and is distributed throughout most parts of East and Southeast Asia (SEA). *R*. *tanezumi* is a common commensal rat in China, where it is also known as the roof rat [1]. *R*. *tanezumi* is a medium-sized rat, presents a more slender profile than *Rattus norvegicus*, and has agile movement and strong climbing abilities. *R*. *tanezumi* often inhabits roofs, wall gaps, and ceilings, thus causing damage to family living areas [2]. This rat is an omnivorous animal that mainly eats plant-based foods, and it is active at night, especially at dusk and dawn. It has a seasonal migration habit. When spring and autumn crops mature, these rats move to the fields and inhabit farmland, where they cause crop damage before harvest [3]. In recent years, the distribution area of *R*. *tanezumi* has expanded significantly because of environmental changes, transportation development, and climate change associated with urbanization [4–6]. In 2002, the Taizhou Entry-Exit Inspection and Quarantine Bureau found live *R*. *tanezumi* inhabiting scrap ships imported from North Korea for the first time. Trains from Tibet to Chongqing were also found to harbor *R*. *tanezumi*. Additionally, a stable population of this rat was detected in Linfen City, Shanxi Province, in 1989 [7]. The distribution area of *R*. *tanezumi* has been expanding. This species is now found in 13 counties of Shanxi Province in areas such as Yuncheng, Changzhi, Jinzhong, Taiyuan, Xinzhou, etc., and its distribution extends into the urban and rural areas of Shanxi. In March 2017, members of this species were caught in the almond forest next to the walnut village of Fenxi County [8]. Because *R*. *tanezumi* has a wide range of activities, it can move back and forth between indoors and outdoors. Traces of its activities are found everywhere, which facilitates the spread of gastrointestinal diseases. These rats harbor ectoparasites, such as mites, and internal parasites, such as protozoa, trematodes, and nematodes. This species is a reservoir host for bacteria, rickettsia, and viruses. *R*. *tanezumi* can spread infectious diseases, such as plague (*Yersinia pestis*), leptospirosis (*Leptospira*), ascariasis, endemic typhus, and hemorrhagic fever with renal syndrome (*Hantaan virus*).

The level of genetic diversity within a species is a result of long-term evolution. Decreased genetic diversity leads to a decline in the ability of organisms to adapt to their environment and survive [9]. Species with higher genetic diversity present stronger adaptability to the environment and more easily expand their distribution. The study of the genetic diversity of *R*. *tanezumi* provides important data for the analysis of the evolutionary potential and future fate of *R*. *tanezumi* and helps reveal the causes and processes underlying *R*. *tanezumi* migration and expansion [10]. The mitochondrial DNA of higher animals is an effective molecular marker that is widely used in phylogenetic research, genetic diversity analysis, and species identification [11, 12]. In 2007, Robins et al. [13] reliably identified *Rattus sp*. with DNA barcoding using cytochrome oxidase I (COI) sequences or tree-based methods using D-loop, cytochrome b and COI sequences.

*Cytb* genes have a moderate evolutionary rate in the mitochondrial genome [14], and a short DNA fragment can contain phylogenetic information from the species to the genus to the class levels. On a certain evolutionary scale, the *Cytb* genes are not severely affected by the saturation effect, and the levels of phylogenetic and genetic differentiation are more suitable for analyzing the differences among species and genera [15, 16]. The *Cytb* gene in animal mitochondria is considered to be a good indicator of the degree of genetic differentiation between related species and within species [17, 18]. Yi et al. [19] studied the *Cytb* gene sequence and showed that the Nile tilapia population in the lower reaches of the Beipan River had rich genetic diversity.

With the development and application of molecular biology, molecular marker technology has been increasingly applied to the genetic study of *R*. *tanezumi* populations. For example, Zhao et al. [20] demonstrated by sequencing the mitochondrial DNA of *R*. *tanezumi* that river barriers have almost no effect on the genetic pattern of this species. Guo et al. [21] suggested that the early dispersal of *R*. *tanezumi* in mainland China was the result of shipping transportation, which led to the subsequent expansion from coastal areas into Central China along the Yangzi River. Furthermore, the linkages between populations in Tibet and Sichuan, revealed by analyzing microsatellite markers and mitochondrial DNA sequences point, to a modern-era introduction via the Chuan-Zang highway rather than the Tea Horse Ancient Road.

This study selected mitochondrial *Cytb* as a molecular marker for 11 geographical populations of *R*. *tanezumi* to study their genetic differentiation, gene communication and phylogenetic tree. Based on the results of these analyses, we aimed to explain the origin, relationship and dispersal of these populations. The degrees of genetic differentiation and genetic diversity of different geographical populations were analyzed, their ecological adaptability was clarified, and their population dynamics were discussed to provide information for the development of effective control strategies for *R*. *tanezumi*.

## Materials and methods

### Ethics statement

This study was approved by the Animal Welfare Provision of the Chinese Academy of Inspection and Quarantine (CAIQ). Approval for the capture of and experimentation on the animals was obtained from CAIQ (approval no. CIAQIHIQ2014-A0003).

### Sampled populations

**Fig 1:**
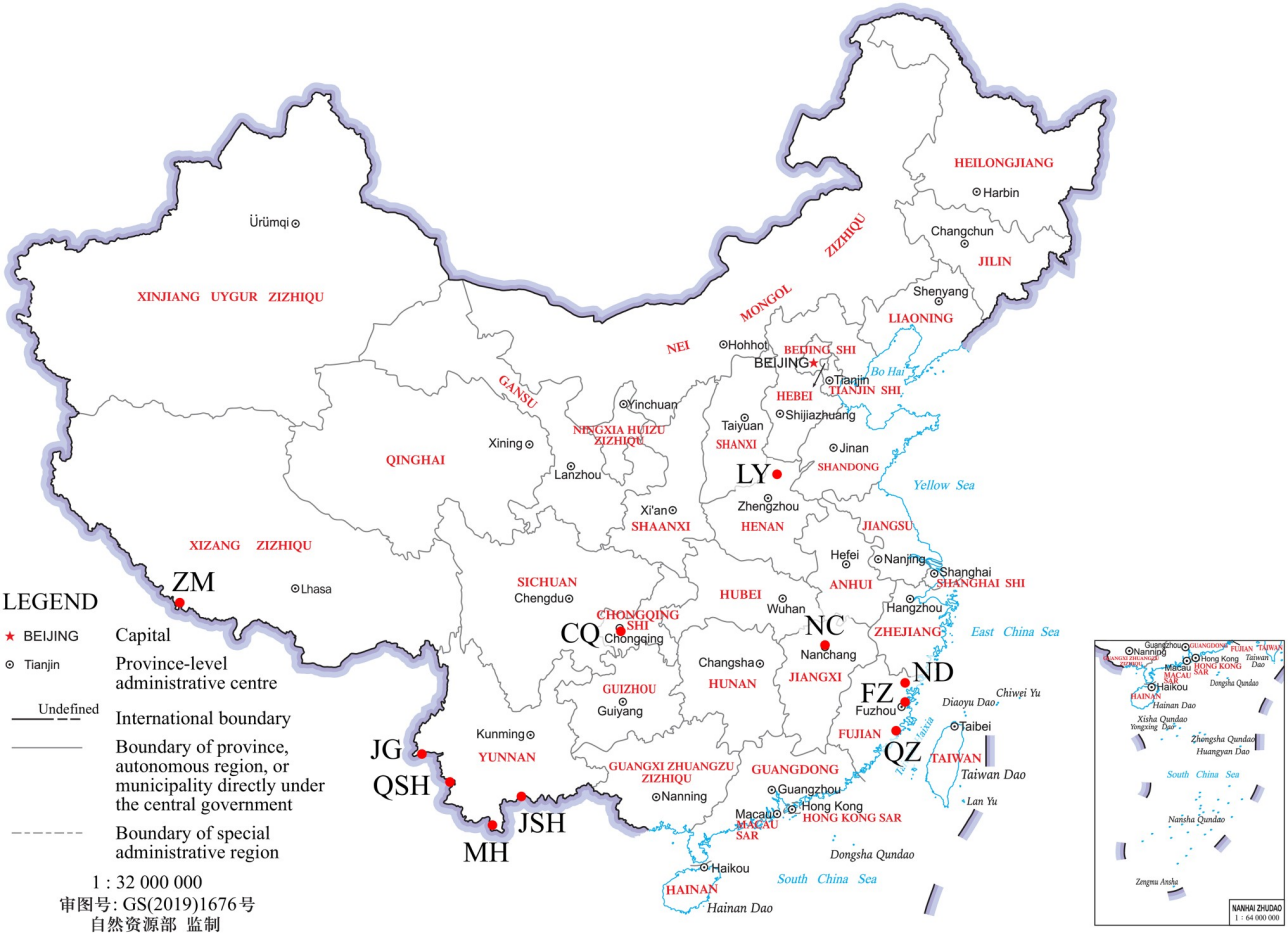
Map showing the distribution of sampling locations.

Samples were collected at 11 locations (N = 5–57 rats per location) in 6 provinces covering the range of *R*. *tanezumi* in China (Fig 1 and Table 1). Samples from Yunnan consisted of specimens pooled from five sites in close proximity (Jiegao (JG), Mohan (MH), Jinshuihe (JSH) and Qingshuihe (QSH)). Samples from Fujian consisted of specimens pooled from three sites in close proximity (Fuzhou (FZ), Ningde (ND), Quanzhou (QZ)). Samples were also obtained from Luoyang Customs, Chongqing Customs and Tibet (ZM). A total of 229 rats were captured. Rats were captured with non-lethal traps in factories, airports and farms after obtaining verbal consent from the owners. All the rats sampled were identified to the species level via external morphological criteria and molecular biology (COI) [22–24]. Five linear measurements were considered: head + body length, tail length, hind-foot length, ear length and head length. All measurements were performed with a ruler (precision 1 mm) except for head length, which was measured with a caliper (precision 0.1 mm). *R*. *tanezumi* is slimmer than *Rattus norvegicus*, and its skull is smaller than that of *R*. *norvegicus*. An important morphological feature for identifying *Rattus losea* is the presence of an obvious dark gray-brown spot in the center of the dorsal forefoot. Additionally, GenBank-sourced sequences from *Mus musculus* and *Volemys kikuchii* were included as outgroups and used as population differentiation time calibration node. The sample locations are detailed in Table 1 and Fig 1.

**Table 1:** Information on *R*. *tanezumi* samples used in the present research.

Locality	Population	Geographic coordinates	Altitude	Habitat	Sample size	*R*. *tanezumi*
Fuzhou, Fujian	FZ	N26°04′38.48″; E119°17′29.67″	84	Indoor	57	10
Ningde, Fujian	ND	N26°39′45.70″; E119°31′20.79″	40	Indoor	15	6
Quanzhou, Fujian	QZ	N24°52′38.10″; E118°40′16.64″	20	Indoor	14	4
Luoyang, Zhengzhou	LY	N34°39′50.36″; E112°25′41.38″	138	Indoor	30	29
Nangchang, Jiangxi	NC	N28°41′10.86″; E115°51′11.03″	47	Indoor	10	10
Chongqing	CQ	N29°33′59.70″; E106°32′48.17″	259	Indoor	10	4
Zhangmu, Xizang	ZM	N27°58′9.24″; E85°57′58.42″	2300	Outdoor	38	21
Jiegao, Yunnan	JG	N23°58′56.54″; E97°53′14.83″	760	Outdoor	20	15
Qingshuihe, Yunnan	QSH	N23°29′42.61″; E98°50′48.30″	468	Outdoor	10	10
Jinshuihe, Yunnan	JSH	N22°33′57.48″; E103°05′56.22″	291	Outdoor	20	17
Mohan, Yunnan	MH	N21°10′58.31″; E101°41′3.11″	923	Outdoor	5	5

### DNA extraction

Rats were selected and transported to the laboratory individually. Compressed carbon dioxide in a gas cylinder was used to euthanize the rats. The captured rats were dissected to obtain their liver tissue, which was collected and stored in 95% ethanol at -80°C until DNA extraction according to the handbook of the Blood/Cell/Tissue Genomic DNA Extraction Kit (TIANGEN).

MtDNA sequence generation DNA extracted from all samples was used as a template; the complete *Cytb* gene (1140 bp) was amplified using primers (L14727-SP 5’-GACAGGAAAAATCATCGTTG-3’; H15915-SP 5’-TTCATTACTGGTTTACAAGAC-3’) and COI (750 bp) was amplified using primers (BatL5310 5’- CCTACTCRGCCATTTTACCTATG-3’; R6036R 5’-ACTTCTGGGTGTCCAAAGAATCA-3’) in a thermocycler. The total volume of the amplification system included 50 μL: 1 μL of template DNA, 1 μL of each primer (10 μM), and 25 μL of Premix Taq (TaKaRa Taq Version 2.0 plus dye), and RNase-Free water to a final volume of 50 μL, which was then mixed. We used a standard 3-step amplification protocol: 95°C for 3 min; 35 cycles of 95°C for 30 s, 50°C (*Cytb*)/55°C (COI) for 30 s, and 72°C for 90 s (*Cytb*)/60 s (COI); 1 cycle of 72°C for 10 min and then held at 4°C. PCR amplification products were subjected to 1.0% agarose gel electrophoresis, Goldview staining and UV gel imager detection. Sequencing was performed by Invitrogen Biotechnology Co., Ltd. Sequences were manually edited in Sequencher v5.3 and aligned with ClustalX 2.0 [25], and these processes were supplemented by manual proofreading.

### Mitochondrial DNA analysis

We used DnaSP v6 [26] and MEGA 7.0 [27] to calculate standard population genetic statistics and test for signals of population expansion based on Tajima’s *D* statistic. Partial deletion of positions with missing data was performed when calculating pairwise nucleotide-based metrics (nucleotide diversity and pairwise number of nucleotide differences). To characterize the population structure of *R*. *tanezumi*, we performed an analysis of molecular variance (AMOVA) of all samples in Arlequin v3.5. We visualized the spatial patterns of mtDNA diversity by computing a TCS haplotype network [28] in PopART using only the more significantly variable *Cytb* genes.

We inferred phylogenies for all samples in a Bayesian framework. First, we used jModelTest 2.1.7 to identify optimal substitution models as the most general model identified by either the Akaike Information Criterion (AIC) or the hierarchical likelihood ratio test. We conducted Bayesian phylogenetic inferences in MrBayes v3.2.3 [29] and BEAST [30] on *Cytb* using the optimal partitioning scheme inferred above. Two independent Markov Chain Monte Carlo (MCMC) analyses composed of 4 Metropolis-coupled chains each (the default) were used to estimate the posterior distributions of the tree topologies, with both analyses run for 1,000,000 generations, sampling every 1000 generations, and discarding the first 25% of samples as burn-in. The convergence of all parameters was assessed in Tracer v1.6.0 [31] by visualizing trace plots and ensuring effective sample sizes of >200.

We used the minimum and maximum time constraints of the mouse–rat divergence from [32] to calibrate the BEAST analysis. These researchers stated that although the location of the split between *Mus* and *Rattus* is somewhat speculative, most current research suggests it occurred early in the evolution of Murinae but was not basal in the divergence of the clade. We used the recommendations of 14 Mya (the oldest record of *Progonomys*, the genus assumed to include the common ancestor of *Mus* and *Rattus*) and 10.4 Mya (based on records of the extinct genus *Karnimata* [33, 34], which is believed to be in the lineage leading to *Rattus*) as upper and lower demarcations, respectively, for the middle 95% of the normally distributed prior divergence. In accordance with all recent molecular and morphological interpretations, the *R*. *tanezumi* clade was enforced relative to that of the vole.

## Results

Through morphological and molecular biology (COI) identification and alignment analysis of these sequences, 131 *R*. *tanezumi* individuals were confirmed among the 229 collected rats, and the nucleotide sequence length was approximately 1120 bp. Overall, the polymorphisms in the *Cytb* region were high, with 126 segregating sites defining 34 haplotypes (S2 Table). The nucleotide diversity indices were highest for the MH population (*π* = 0.0098) in Yunnan but lowest for the ND population (*π* = 0.0000) in Fujian. Significant differences were not observed in the neutral detection of Tajima’s *D* statistic (Tajima’s *D*: -0.59483, P > 0.10) for all geographical populations.

Table 2 shows that the genetic differentiation coefficient *Fst* was between 0.00000 and 0.99207. The gene flow *Nm* was between 0.00 and 51.74. The *Fst* and *Nm* values between FZ and ND were 0.00741 and 33.50, respectively, while the *Fst* and *Nm* between QZ and ND were 0.33333 and 0.50, respectively. The *Fst* values between the *R*. *tanezumi* population of ZM and other geographic populations were greater than 0.80, whereas the Nm values were smaller than 0.50. The *Fst* and *Nm* between FZ and NC, FZ and LY, and FZ and CQ were 0.04884 and 4.87, 0.00486 and 51.74, and 0.00635 and 39.13, respectively. The *Fst* and *Nm* between QZ and NC, QZ and LY, and QZ and CQ were 0.20486 and 0.97, 0.09764 and 2.31, and 0.22222 and 0.88, respectively. The *Fst* of ND and NC, ND and LY, ND and CQ was 0.18107, 0.00000 and 0.00000, respectively. The *Fst* and *Nm* between MH and JSH were 0.02541 and 10.09, those between MH and QSH were 0.00604 and 41.67, and those between MH and JG were 0.11466 and 1.93, respectively. The *Fst* and *Nm* values between JG and JSH were 0.14863 and 1.43, and those between JG and QSH were 0.05163 and 4.59, respectively.

**Table 2:** Gene flow (*Nm*; above the diagonal) and differentiation coefficient (*Fst*; below the diagonal) between populations.

	FZ	ND	QZ	NC	LY	CQ	JSH	MH	QSH	JG	ZM
FZ		33.5	2.56	4.87	51.74	39.13	0.74	0.79	0.45	0.34	0.01
ND	0.00741		0.50	1.13	——	——	0.40	0.47	0.27	0.23	0.00
QZ	0.08889	0.33333		0.97	2.31	0.88	0.41	0.48	0.28	0.24	0.00
NC	0.04884	0.18107	0.20486		1.83	1.26	0.69	0.69	0.43	0.34	0.01
LY	0.00486	0.00000	0.09764	0.12048		——	0.53	0.61	0.37	0.30	0.01
CQ	0.00635	0.00000	0.22222	0.16573	0.0000		0.42	0.50	0.29	0.24	0.00
JSH	0.25364	0.38462	0.38147	0.26690	0.32004	0.37244		10.09	6.63	1.43	0.03
MH	0.24034	0.34524	0.34457	0.26561	0.29118	0.33526	0.02541		41.67	1.93	0.03
QSH	0.35534	0.47674	0.46886	0.36628	0.40575	0.46328	0.03633	0.00604		4.59	0.02
JG	0.42101	0.51880	0.51008	0.42097	0.45664	0.50631	0.14863	0.11466	0.05163		0.03
ZM	0.96438	0.99207	0.98590	0.95190	0.96950	0.98740	0.90620	0.89479	0.90934	0.90395	

Due to the close geographical location and high degree of gene exchange between the FZ and ND *R*. *tanezumi* populations, we considered the *R*. *tanezumi* populations in these two regions as a group, and similarly grouped the JSH, MH and QSH populations; thus, we divided the 11 *R*. *tanezumi* populations into 8 groups. The AMOVA results (Table 3) showed that the percentage of variation (20.48%) among populations of *R*. *tanezumi* was smaller than the percentage of variation among 8 groups (78.02%), and the percentage of variation among populations within groups was 1.50%.

**Table 3:** Analysis of Molecular Variance (AMOVA) among 11 populations of *R*. *tanezumi*.

Source of Variation	*df*	Sum of Squares	Variance Components	Percentage of Variation (%)
Among groups	7	995.491	8.94599 Va	78.02
Among populations within groups	3	11.618	0.17225 Vb	1.50
Within populations	119	279.375	2.34769 Vc	20.48
Total	129	1286.485	11.46593	100.00

**Fig 2:**
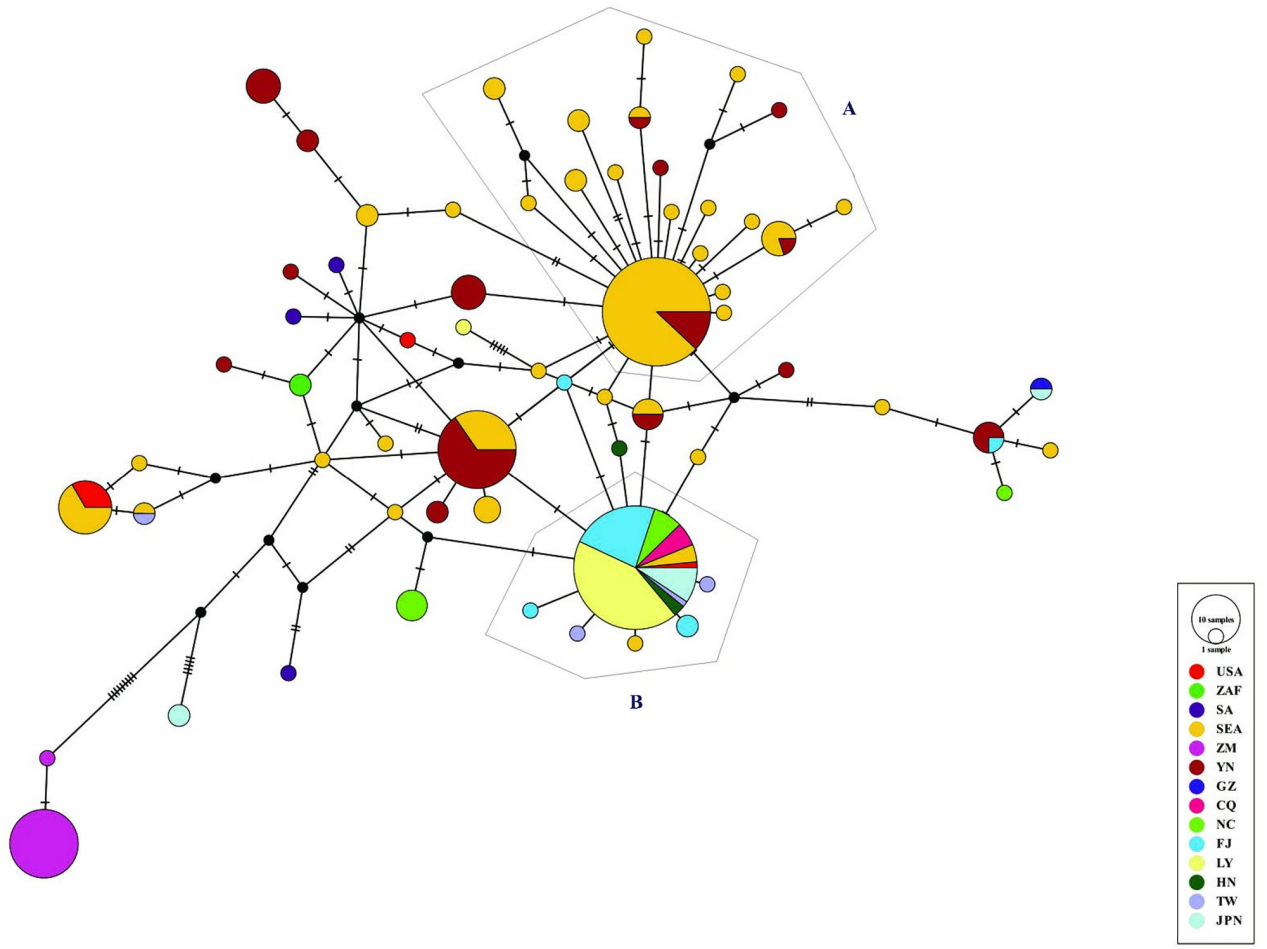
Haplotype network of *R*. *tanezumi* based on the *Cytb* gene of mtDNA. See S1 Table for a full description of *Cytb* sequences.

Regarding the network analyses, the *R*. *tanezumi* clade consisted of 63 unique haplotypes. There were fifteen unique Yunnan *R*. *tanezumi* haplotypes, of which five haplotypes were either shared with or differed by only a single mutation from SEA haplotypes and were removed from ZM *R*. *tanezumi* haplotypes (Fig 2). The two *R*. *tanezumi* haplotypes recovered from ZM were not shared outside of ZM and formed a closely related cluster that also contained two Bangladesh and one South Africa *R*. *tanezumi* haplotype. Haplotypes from other parts of China were shared, and these were shared with Japan, SEA, and the United States.

According to the TCS network (Fig 2), H2 included Hainan, Taiwan, Fujian, NC, LY, CQ, SEA and Japan, which had the highest frequency, at 24.34% (65/267), of all tested individuals; H5 and H16 included Yunnan and SEA, accounting for 9.74% (26/267) and 18.73% (50/267) of the total individuals, respectively. The two haplotypes that characterized the *R*. *tanezumi* population from ZM were not shared with those from other geographic populations. *R*. *tanezumi* Hap62 accounted for 7.49% (20/267) of the total individuals. Two peripheral star-like clusters are present in the *R*. *tanezumi* network (labelled A and B of Fig 2). Cluster A comprises haplotypes from Southeast Asia (Thailand, Laos) and Yunnan (MH, JG). The central haplotype of Cluster A is represented in rats from Thailand, Laos and JSH. Cluster B mainly comprises haplotypes from the western Pacific margin, including Vietnam, Fujian and Taiwan. The central haplotype of Cluster B is represented in rats from Taiwan, Fujian, Hainan, NC, LY, CQ, and also from Japan, the United States, Vietnam and Myanmar.

**Fig 3:**
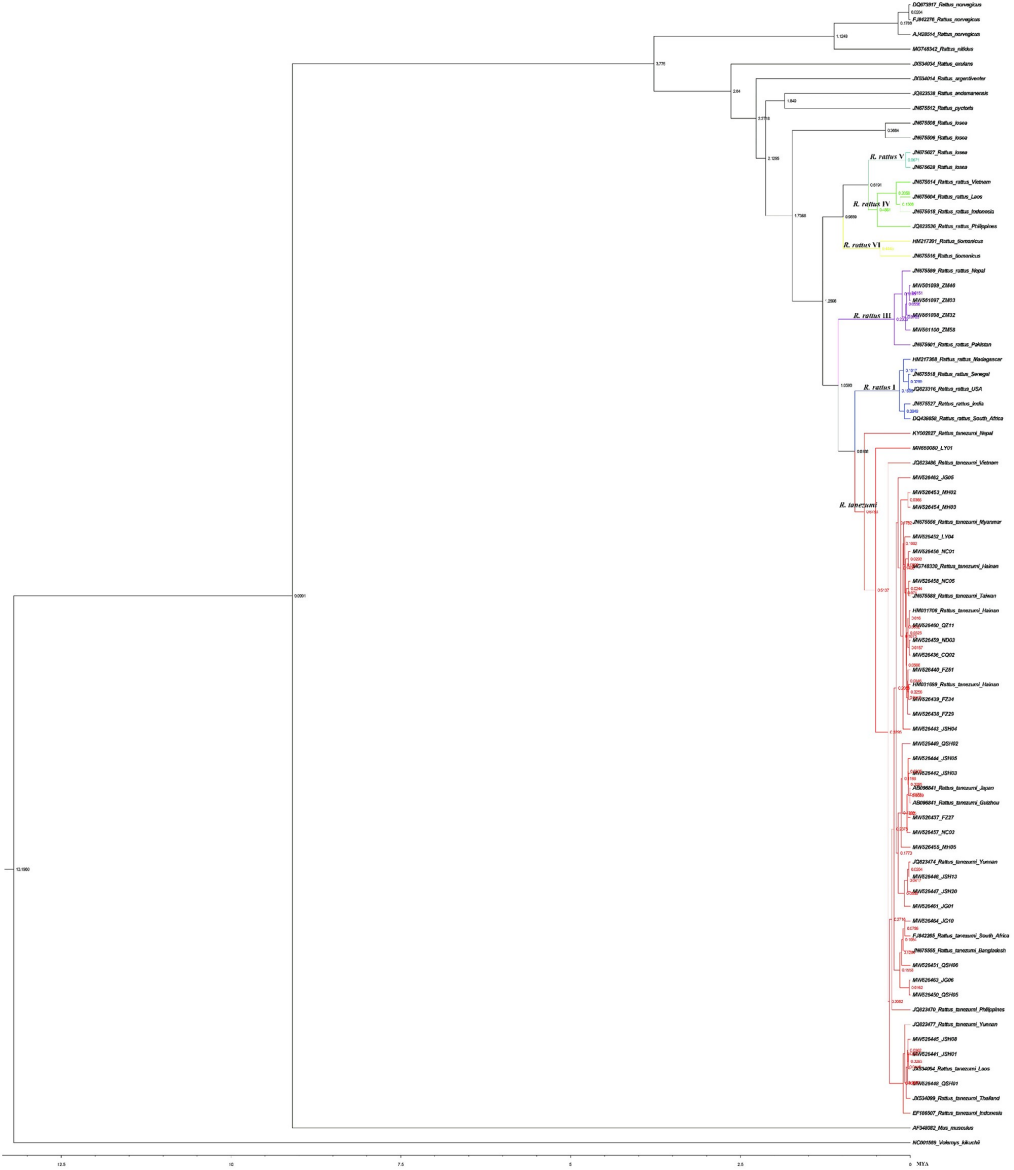
Phylogenetic tree produced with Bayesian inference using MCMC. See S1 Table for a full description of *Cytb* sequences.

First, we performed a phylogenetic analysis of 427 *Cytb* gene sequences (including 296 downloaded from the NCBI database and 131 *Cytb* gene sequences obtained in this study). The combined data set of carefully screened sequences derived from all available sequences in GenBank along with our current data resulted in a robust maximum likelihood phylogenetic analysis of 78 *Cytb* gene sequences. The trees estimated by ML and Bayesian inference from the *R*. *tanezumi Cytb* dataset had the same topology (Fig 3). *Cytb* sequences were generated from 34 *R*. *tanezumi*, excluding those with the same sequences (S3 Table). In Tracer, the effective sample size (ESS) value was greater than 200, indicating that the parameters in MrBayes and BEAST were reasonable; thus, the divergence time tree of *R*. *tanezumi* was finally obtained.

The Bayesian phylogenetic analysis of *Cytb* placed all of the sampled *R*. *tanezumi* and clustered *R*. *rattus* in six distinct and statistically supported clades corresponding to lineages I, II, III, IV, V and VI from Aplin et al. [22] and Lack et al. [35]. In clade II, the time of differentiation of *R*. *tanezumi* was earliest in Nepal, estimated at 0.68 Mya. The next was LY (LY01), at approximately 0.52 Mya, followed by the Philippines, at 0.33 Mya, and Yunnan (JSH01, JSH08 and QSH01), at about 0.31 Mya; finally *R*. *tanezumi* from the rest of China, Japan, South Africa, Madagascar, and Bangladesh diverged 0.27 Mya. With the exception of a subset of individuals from ZM that were closely related to populations from Nepal and Pakistan, which belonged to the *R*. *rattus* III clade (highlighted in purple in Fig 3) and diverged 1.06 Mya, all individuals belonged to the *R*. *rattus* II clade (highlighted in red in Fig 3).

## Discussion

In the past, *R*. *tanezumi* was mainly distributed in the Yangtze River Basin and areas to the south. Outside of China, populations were mainly distributed in parts of SEA, such as India, Myanmar, Laos, and Vietnam. Our test randomly selected 6 provinces in China (Tibet, Yunnan, Fujian, CQ, LY, and NC). ZM Port is located in the southern foothills of the Himalayas, and it is the largest border trade center port in Tibet. The trade routes radiate to Tibet and neighboring provinces and regions and to Nepal and neighboring countries and regions. The main channel for economic and cultural exchange between China and the South Asian subcontinent is the largest open port. The port has a dense population flow, frequent trade activity, good weather, and obvious traces of rodent activity. The climate type in Yunnan is complex, and the terrain is high in the north and low in the south. Due to the influence of topography and different weather systems, the zonal distribution of temperature in the province frequently presents unique phenomena, such as "hot in the north and cool in the south". Moreover, the distribution of precipitation seasonally and regionally is extremely uneven. Therefore, four regions in Yunnan Province were selected from north to south (JG, QSH, MH, and JSH). The geography of Fujian is characterized by mountains and sea, and it has a forest coverage rate of 65.95%, ranking first in the country. FZ, ND and QZ are all coastal cities with suitable temperatures, and they host large populations of *R*. *lose*, *R*. *norvegicus* and *R*. *tanezumi*. The inland cities of CQ, LY and NC have developed road and railway transportation networks and abundant transportation means, which may represent a method for the abnormal spread of *R*. *tanezumi*.

The level of genetic diversity within a species is closely related to its evolutionary potential and ability to adapt to the environment. Higher genetic diversity provides a great advantage for species to survive and reproduce under environmental stress [36, 37]. Haplotype diversity (*H*) and nucleotide diversity (*π*) are two important indicators of genetic diversity [38, 39]. Overall, the *R*. *tanezumi* populations of Yunnan showed a pattern of high haplotype diversity and high nucleotide diversity, indicating that a large and stable population had evolved over a long period of time or that secondary contact occurred between different populations [40]. There was abundant haplotype diversity in each geographic population of *R*. *tanezumi* in Yunnan, indicating that the *Cytb* genes of *R*. *tanezumi* had a high rate of polymorphism, which suggested that they had strong survivability in changing environments. This characteristic might be related to various factors, such as their biological characteristics. In contrast, the genetic diversity of the populations in the present research showed that the ND population, LY population and Tibet populations had lower genetic variability (S2 Table). The low genetic diversity of ND and LY could be attributed to the founder effect. The low genetic diversity of populations from Tibet (ZM) could only be explained as a result of the founder effect as there were no rodent control measures in Tibet. The recent demographic expansion of these populations was also supported the results of Tajima’s *D* statistics.

Tajima’s *D* test is a neutral test method based on intraspecies polymorphism. In recent years, *R*. *tanezumi* has been controlled almost every year in either urban or rural areas because it is a host of pathogens that cause plague (*Y*. *pestis*), hemorrhagic fever (*H*. *virus*) and leptospirosis (*Leptospira*). As a result, Tajima’s *D* statistics of all populations were negative and not significant. A negative D value indicated that there was a slight deleterious mutation in the purifying selection or separation of the *R*. *tanezumi* population, which may also be caused by population expansion [41, 42]. Guo et al. [21] performed Tajima’s *D* and Fu & Li’s tests on the D-loop and COI genes of 12 *R*. *tanezumi* populations and found recent demographic expansion of these populations, which was consistent with our findings. COI is a widely used DNA barcoding method that shows sufficient resolution and stability in biological species and is suitable for species identification. Therefore, we used the COI gene to identify the species of the rats we captured. The *Cytb* gene evolution rate is moderate, and the gene contains a large amount of genetic information and is particularly suitable for analyzing the genetic evolution among individuals of the same species. *Cytb* is more suitable for the construction of phylogenetic trees to study the geographic distribution of species [43–47]. We speculated that there might be differences in the genetic characteristics of *R*. *tanezumi* populations with different mitochondrial genes.

In the *Fst* and *Nm* analyses of *R*. *tanezumi* populations from 11 regions (Table 2), FZ and ND had a low degree of genetic differentiation, as did JSH, MH and QSH, probably because of their relatively close proximity. ZM and other areas of *R*. *tanezumi* populations had a high degree of genetic differentiation, probably owing to the high altitude of ZM, which is located on the slope of the southern valley of the middle section of the Himalayas on the Sino-Nigerian border, and its environment might affect the genetic evolution of *R*. *tanezumi*. The population of *R*. *tanezumi* in the MH and JSH areas had a relatively high degree of gene exchange with populations in the other areas, but the populations in the QSH and JG areas had a relatively low degree of gene exchange with populations in other areas. Eastern Yunnan is characterized by undulating low mountains and hills, and various types of karst landforms have developed; thus, communication between *R*. *tanezumi* populations had relatively less influence than other factors. Western Yunnan is a longitudinal valley area of the Hengduan Mountains. Alpine valleys have relatively high elevation differences and steep terrain, which to a certain extent hinders communication between *R*. *tanezumi* populations. In the southwestern border area, the terrain gradually flattens, and the populations of MH and QSH had more gene exchange than did the MH and JSH *R*. *tanezumi* populations. It is speculated that the *R*. *tanezumi* population in southern Yunnan spread to western Yunnan and that the southwestern *R*. *tanezumi* populations experienced close gene communication. A riverine barrier hypothesis may also be feasible. MH and JSH are separated by the Red River. As a geographical barrier, the river may hinder the migration and gene flow of terrestrial animals, thereby causing population differentiation and promoting the formation of population patterns [48–50]. Furthermore, the results of AMOVA confirmed that the variation in different groups was high, whereas the variation among the populations within groups was lower, so the genetic differences mainly occur among geographic populations, indicating that the degree of gene exchange within *R*. *tanezumi* populations was greater than that between populations.

According to the minimum spanning haplotype network (Fig 2), the haplotypes in the local populations presented a mixed distribution pattern but with a fairly obvious geographical distribution. The haplotype from ZM was far from the other haplotypes, and the evolutionary relationship presents multiple stellate radiations, revealing that the *R*. *tanezumi* population had undergone local expansion many times in history, which is also supported by the results of Tajimaʹs *D* test. In addition, the *R*. *tanezumi* populations of FZ, ND, QZ, NC, LY and CQ had shared haplotypes, indicating that these 6 *R*. *tanezumi* populations were closely related. Similarly, the *R*. *tanezumi* populations of JSH, QSH, MH and JG were relatively close. Exclusive haplotypes that occurred independently in each geographic population indicated that there was a certain degree of genetic differentiation in each geographic population as well as a certain degree of gene communication [51].

Zhou [52] used four gene fragments of *Cytb*, COI, 16S and IRBP from animal samples to construct a phylogenetic tree, and he found that the topological structure of the phylogenetic tree of each gene fragment was inconsistent, and the phylogenetic tree constructed by a single gene sequence had differences in topological structure. *Cytb* and IRBP appeared to be more suitable for constructing a single-species phylogenetic tree than COI and 16S. However, the genetic analysis by Pages et al. [53] revealed discordance between the mitochondrial and nuclear data. In this study, we selected the mitochondrial *Cytb* gene to analyze the genetic characteristics of the *R*. *tanezumi* populations in different regions and obtained relatively good results. The Bayesian phylogenetic analysis of cytochrome b placed all of the sampled *R*. *tanezumi* and gathered *R*. *rattus* in six distinct and statistically supported clades corresponding to lineages I, II, III, IV, V and VI from Aplin et al. [22] and Lack et al. [35]. And we learned that populations with Lineages II, III and IV would all be regarded as *R*. *tanezumi* under current taxonomy from the study of Aplin et al. From the analysis of the Bayesian evolutionary tree, *R*. *tanezumi* populations from the same area were generally preferentially clustered together, followed by clusters with relatively close geographical locations, and included the cross-branching of branches in various regions, with a fairly obvious geographical distribution. The phylogenetic tree showed that the *R*. *tanezumi* population in ZM was closely related to those in Nepal and Pakistan. Compared with populations in other regions, ZM *R*. *tanezumi* clustered preferentially with those from Nepal and Pakistan, which belonged *R*. *rattus* III and were independent of samples from other regions. We speculate that *R*. *tanezumi* in ZM also belong to the *R*. *rattus* III clade, which differentiated earlier than *R*. *tanezumi* in other parts of China (belonging to the *R*. *rattus* II clade). The reason might be that the ZM port provided a connection to Nepal and Pakistan, and the relatively close distance was conducive to the spread of the *R*. *tanezumi* population. Similarly, the *R*. *tanezumi* population in Yunnan was closely related to those in Thailand, Laos, the Philippines, Indonesia, Myanmar and Bangladesh. In southwestern Yunnan, most of the JG *R*. *tanezumi* samples were closely related to those from QSH and were relatively geographically close to each other, which was consistent with the close genetic exchange between the two groups of *R*. *tanezumi* shown in Table 3. In recent decades, the northward expansion of *R*. *tanezumi* has become obvious in North China, such as in Shanxi and Qinghai [54, 55], which suggests that modern rapid transportation has provided additional opportunities for long-distance dispersal of *R*. *tanezumi*. Although the geographical locations of ZM and LY are far apart, we speculate that *R*. *tanezumi* might have dispersed between them. Some of the populations of *R*. *tanezumi* in FZ, ND, QZ, NC, LY and CQ were closely related, which roughly confirmed the results indicated by the haplotype network diagram. In southeastern Yunnan, the populations of *R*. *tanezumi* in JSH and MH were more closely related to the populations in southeastern China (FZ, ND, QZ, and NC) and inland areas (CQ and LY) than to those in other areas of Yunnan, showing that *R*. *tanezumi* might be more inclined to spread from the southeast of Yunnan to the interior of China.

Hal postulated that beginning in the early Pliocene (~5 Mya), a combination of emergent small islands and changing sea levels led to changes in the spread of rodents [56]. Dating analyses indicated that the *Cytb* gene in these lineages initially diverged about 1 Mya, with the earliest at 1.06 Mya in ZM, followed by *R*. *rattus* I/*R*. *tanezumi* divergence approximately 0.8 Mya. We discovered *R*. *tanezumi* populations in ZM that had differentiated earlier than those in other regions of China. It could be assumed that ZM and the adjacent area of SEA could be regarded as the origin of *R*. *tanezumi*. Interestingly, the differentiation time of *R*. *tanezumi* in LY was earlier than those in Yunnan and other regions. There are likely many mechanisms for the eastward spread of *R*. *tanezumi* from ZM to inland China. One possibility that all the *R*. *tanezumi* caught in LY were caught at the airport, and *R*. *tanezumi* in other areas spread to LY through transportation modes, such as airplanes.

In summary, *R*. *tanezumi* may have originated in ZM and adjacent areas, spread to Yunnan, and then spread from the southeast of Yunnan inland or directly eastward from ZM to inland China. The dispersal route needs further study. *R*. *tanezumi* may have also spread through transportation networks, such as highways and railways. Our study and that of Robins et al. [34] are based only on the mitochondrial genome and *Cytb*, thus are subject to intrinsic biases related to maternal inheritance and organelle location [57, 58], resulting in partial differences in results, but no significant differences were found with respect to the margins of error. In addition, two sources of uncertainty remain concerning the inferred timescale [22]. Firstly, it is now well known that molecular rates change with time [59–61], and a single depth calibration point at the interspecific level may not provide optimal accuracy for estimation of the intraspecific level. On different time scales, only a combination of several calibration points can significantly improve dating accuracy [62, 63]. Therefore, information from multiple markers needs to be used to obtain a more reliable estimate of the phylogenetic diversification time of this recently evolved population.

## Supporting information

S1 Table Information about the additional samples used in Figs 2 and 3. (PDF)

S2 Table Statistics of *Cytb* gene diversity in *R*. *tanezumi*. (PDF)

S3 Table Statistics for the same sequences from the 131 *Cytb* sequences. (PDF)

S1 File Sequences. (TXT)
