# Supplementary material for: Genetic differentiation of geographic populations of Rattus tanezumi based on the mitochondrial Cytb gene
Source: PLoS One. 2021 Mar 18;16(3):e0248102. doi: 10.1371/journal.pone.0248102 (PMC7971478; doi:10.1371/journal.pone.0248102)
Supplement: S1 Table — (PDF) [file pone.0248102.s001.pdf]

S1 Table. Information Regarding the Samples Used in this Study.

Country	Taxon	Accession No.
—	*Volemys kikuchii*	AF348082
—	*Mus musculus*	NC001569
—	*Rattus andamanensis*	JQ823538
—	*Rattus argentiventer*	JX534014
—	*Rattus exulans*	JX534034
—	*Rattus losea*	JN675508
—	*Rattus losea*	JN675506
—	*Rattus losea*	JN812628
—	*Rattus losea*	JN812627
—	*Rattus norvegicus*	DQ673917
—	*Rattus norvegicus*	AJ428514
—	*Rattus norvegicus*	FJ842276
—	*Rattus nitidus*	MG748342
—	*Rattus pyctoris*	JN675512
—	*Rattus tiomanicus*	JN675516
—	*Rattus tiomanicus*	HM217391
Nepal	*Rattus rattus*	JN675599
Pakistan	*Rattus rattus*	JN675601
India	*Rattus rattus*	JN675527
Senegal	*Rattus rattus*	JN675518
USA	*Rattus rattus*	JQ823316
South Africa	*Rattus rattus*	DQ439858
Madagascar	*Rattus rattus*	HM217368
Philippines	*Rattus rattus*	JQ823536
Vietnam	*Rattus rattus*	JN675614
Indonesia	*Rattus rattus*	JN675618
Laos	*Rattus rattus*	JN675604
Guizhou	*Rattus tanezumi*	AB096841
Hainan	*Rattus tanezumi*	HM031699
Hainan	*Rattus tanezumi*	HM031706
Hainan	*Rattus tanezumi*	MG748330
Yunnan	*Rattus tanezumi*	JQ823474
Yunnan	*Rattus tanezumi*	JQ823475
Yunnan	*Rattus tanezumi*	JQ826476
Yunnan	*Rattus tanezumi*	JQ823477
Taiwan	*Rattus tanezumi*	JN675591
Taiwan	*Rattus tanezumi*	JN675590
Taiwan	*Rattus tanezumi*	JN675589
Taiwan	*Rattus tanezumi*	JN675588
Japan	*Rattus tanezumi*	EU273712

Japan	*Rattus tanezumi*	JN675593
Japan	*Rattus tanezumi*	JN675592
Japan	*Rattus tanezumi*	EF186508
Japan	*Rattus tanezumi*	AB211040
Japan	*Rattus tanezumi*	AB211041
Japan	*Rattus tanezumi*	AB211042
Japan	*Rattus tanezumi*	AB211043
Japan	*Rattus tanezumi*	AB096841
Bangladesh	*Rattus tanezumi*	JN675555
Bangladesh	*Rattus tanezumi*	JN675554
South Africa	*Rattus tanezumi*	FJ842265
South Africa	*Rattus tanezumi*	DQ439850
USA	*Rattus tanezumi*	JQ823492
USA	*Rattus tanezumi*	JQ823493
USA	*Rattus tanezumi*	JQ823472
USA	*Rattus tanezumi*	JN675598
USA	*Rattus tanezumi*	JN675597
USA	*Rattus tanezumi*	JN675596
Thailand	*Rattus tanezumi*	JX534118
Thailand	*Rattus tanezumi*	JX534117
Thailand	*Rattus tanezumi*	JX534116
Thailand	*Rattus tanezumi*	JX534115
Thailand	*Rattus tanezumi*	JX534114
Thailand	*Rattus tanezumi*	JX534113
Thailand	*Rattus tanezumi*	JX534112
Thailand	*Rattus tanezumi*	JX534111
Thailand	*Rattus tanezumi*	JX534110
Thailand	*Rattus tanezumi*	JX534109
Thailand	*Rattus tanezumi*	JX534108
Thailand	*Rattus tanezumi*	JX534107
Thailand	*Rattus tanezumi*	JX534106
Thailand	*Rattus tanezumi*	JX534105
Thailand	*Rattus tanezumi*	JX534104
Thailand	*Rattus tanezumi*	JX534103
Thailand	*Rattus tanezumi*	JX534101
Thailand	*Rattus tanezumi*	JX534100
Thailand	*Rattus tanezumi*	JX534099
Thailand	*Rattus tanezumi*	JX534098
Thailand	*Rattus tanezumi*	JX534097
Thailand	*Rattus tanezumi*	JX534096
Thailand	*Rattus tanezumi*	JX534094
Thailand	*Rattus tanezumi*	JX534092
Thailand	*Rattus tanezumi*	JX534091
Thailand	*Rattus tanezumi*	JX534090

Thailand	*Rattus tanezumi*	JX534089
Thailand	*Rattus tanezumi*	JX534087
Thailand	*Rattus tanezumi*	JX534086
Thailand	*Rattus tanezumi*	JX534084
Thailand	*Rattus tanezumi*	JX534083
Thailand	*Rattus tanezumi*	JX534082
Thailand	*Rattus tanezumi*	JX534081
Thailand	*Rattus tanezumi*	JX534080
Thailand	*Rattus tanezumi*	JX534079
Thailand	*Rattus tanezumi*	JX534078
Thailand	*Rattus tanezumi*	JX534077
Thailand	*Rattus tanezumi*	JX534075
Thailand	*Rattus tanezumi*	JX534074
Thailand	*Rattus tanezumi*	JX534073
Thailand	*Rattus tanezumi*	JX534072
Thailand	*Rattus tanezumi*	JX534071
Thailand	*Rattus tanezumi*	JX534070
Thailand	*Rattus tanezumi*	JX534069
Thailand	*Rattus tanezumi*	JX534068
Thailand	*Rattus tanezumi*	JX534067
Thailand	*Rattus tanezumi*	JX534066
Thailand	*Rattus tanezumi*	HM217467
Thailand	*Rattus tanezumi*	HM217410
Thailand	*Rattus tanezumi*	HM217456
Thailand	*Rattus tanezumi*	HM217407
Thailand	*Rattus tanezumi*	HM217466
Thailand	*Rattus tanezumi*	HM217426
Thailand	*Rattus tanezumi*	HM217457
Thailand	*Rattus tanezumi*	HM217436
Thailand	*Rattus tanezumi*	HM217430
Thailand	*Rattus tanezumi*	HM217452
Thailand	*Rattus tanezumi*	HM217398
Thailand	*Rattus tanezumi*	HM217371
Thailand	*Rattus tanezumi*	HM217438
Thailand	*Rattus tanezumi*	JN675562
Thailand	*Rattus tanezumi*	JN675561
Thailand	*Rattus tanezumi*	JN675560
Thailand	*Rattus tanezumi*	JN675559
Laos	*Rattus tanezumi*	JX534065
Laos	*Rattus tanezumi*	JX534064
Laos	*Rattus tanezumi*	JX534063
Laos	*Rattus tanezumi*	JX534062
Laos	*Rattus tanezumi*	HM217475
Laos	*Rattus tanezumi*	HM217480

Laos	*Rattus tanezumi*	JN675571
Laos	*Rattus tanezumi*	JN675570
Laos	*Rattus tanezumi*	JN675569
Laos	*Rattus tanezumi*	JN675568
Laos	*Rattus tanezumi*	JN675566
Laos	*Rattus tanezumi*	JN675565
Laos	*Rattus tanezumi*	JN675564
Laos	*Rattus tanezumi*	JN675563
Viet Nam	*Rattus tanezumi*	JQ823483
Viet Nam	*Rattus tanezumi*	JQ823479
Viet Nam	*Rattus tanezumi*	JQ823486
Viet Nam	*Rattus tanezumi*	JQ823462
Viet Nam	*Rattus tanezumi*	JQ823494
Viet Nam	*Rattus tanezumi*	JQ823489
Viet Nam	*Rattus tanezumi*	AB355901
Viet Nam	*Rattus tanezumi*	JN675573
Viet Nam	*Rattus tanezumi*	JN675572
Viet Nam	*Rattus tanezumi*	JN675574
Philippines	*Rattus tanezumi*	JQ823491
Philippines	*Rattus tanezumi*	JQ823471
Philippines	*Rattus tanezumi*	JQ823470
Philippines	*Rattus tanezumi*	JQ823469
Philippines	*Rattus tanezumi*	JN675587
Philippines	*Rattus tanezumi*	JN675586
Philippines	*Rattus tanezumi*	JN675585
Indonesia	*Rattus tanezumi*	EF186491
Indonesia	*Rattus tanezumi*	EF186493
Indonesia	*Rattus tanezumi*	EF186494
Indonesia	*Rattus tanezumi*	EF186507
Indonesia	*Rattus tanezumi*	JN675582
Indonesia	*Rattus tanezumi*	JN675580
Myanmar	*Rattus tanezumi*	JN675558
Myanmar	*Rattus tanezumi*	JN675557
Myanmar	*Rattus tanezumi*	JN675595
Nepal	*Rattus tanezumi*	KY002827
Fuzhou	*Rattus tanezumi*	MW526437
Fuzhou	*Rattus tanezumi*	MW526438
Fuzhou	*Rattus tanezumi*	MW526439
Fuzhou	*Rattus tanezumi*	MW526440
Ningde	*Rattus tanezumi*	MW526459
Quanzhou	*Rattus tanezumi*	MW526460
Nanchang	*Rattus tanezumi*	MW526456
Nanchang	*Rattus tanezumi*	MW526457
Nanchang	*Rattus tanezumi*	MW526458

Luoyang	*Rattus tanezumi*	MN660080
Luoyang	*Rattus tanezumi*	MW526452
Chongqing	*Rattus tanezumi*	MW526436
Jinshihe	*Rattus tanezumi*	MW526441
Jinshihe	*Rattus tanezumi*	MW526442
Jinshihe	*Rattus tanezumi*	MW526443
Jinshihe	*Rattus tanezumi*	MW526444
Jinshihe	*Rattus tanezumi*	MW526445
Jinshihe	*Rattus tanezumi*	MW526446
Jinshihe	*Rattus tanezumi*	MW526447
Qingshuhe	*Rattus tanezumi*	MW526448
Qingshuhe	*Rattus tanezumi*	MW526449
Qingshuhe	*Rattus tanezumi*	MW526450
Qingshuhe	*Rattus tanezumi*	MW526451
Mohan	*Rattus tanezumi*	MW526453
Mohan	*Rattus tanezumi*	MW526454
Mohan	*Rattus tanezumi*	MW526455
Jiegao	*Rattus tanezumi*	MW526461
Jiegao	*Rattus tanezumi*	MW526462
Jiegao	*Rattus tanezumi*	MW526463
Jiegao	*Rattus tanezumi*	MW526464
Zhangmu	*Rattus tanezumi*	MW561097
Zhangmu	*Rattus tanezumi*	MW561098
Zhangmu	*Rattus tanezumi*	MW561099
Zhangmu	*Rattus tanezumi*	MW561100
