# Supplementary material for: Genetic differentiation of geographic populations of Rattus tanezumi based on the mitochondrial Cytb gene
Source: PLoS One. 2021 Mar 18;16(3):e0248102. doi: 10.1371/journal.pone.0248102 (PMC7971478; doi:10.1371/journal.pone.0248102)
Supplement: S2 Table — (PDF) [file pone.0248102.s002.pdf]

S2 Table. Statistics of *Cytb* Gene Diversity in *R. tanezumi*.

Location	Segregating Sites(*S*)	Haplotypes(*H*)	Haplotype diversity(*Hd*)	Average number of differences(*K*)	Nucleotide diversity(π)
FZ	14	5/10	0.6667	2.9778	0.0027
ND	0	1/6	0.0000	0.0000	0.0000
QZ	1	2/4	0.6667	0.6667	0.0006
NC	17	4/10	0.7333	4.4222	0.0040
LY	35	3/29	0.1355	2.4138	0.0022
CQ	1	2/4	0.5000	0.5000	0.0004
JSH	29	8/17	0.8824	9.4118	0.0084
QSH	22	4/10	0.7778	9.0000	0.0080
MH	22	4/5	0.9000	11.0000	0.0098
JG	31	6/15	0.7619	9.7524	0.0087
ZM	7	4/21	0.2714	0.8381	0.0007
