# Supplementary material for: Genetic differentiation of geographic populations of Rattus tanezumi based on the mitochondrial Cytb gene
Source: PLoS One. 2021 Mar 18;16(3):e0248102. doi: 10.1371/journal.pone.0248102 (PMC7971478; doi:10.1371/journal.pone.0248102)
Supplement: S3 Table — (PDF) [file pone.0248102.s003.pdf]

S3 Table. Statistics of the same sequences in 131 *Cytb* sequences.

Sequence number	The same sequences	Numbers
QZ11	QZ13	1
NC01	NC02, NC04, NC10	3
ND03	FZ03, FZ12, FZ14, FZ26, FZ28, FZ53, ND04, ND06, ND07, ND11, QZ12, QZ14, NC06, NC07, NC08, NC09, LY02, LY03, LY05, LY07, LY08, LY09, LY10, LY11, LY12, LY13, LY14, LY17, LY18, LY20, LY21, LY22, LY23, LY24, LY25, LY26, LY27, LY28, LY29, LY30, CQ01, CQ04,CQ05	43
JSH01	JSH11	1
JSH05	JSH10	1
JSH04	JSH07, JSH16, JSH17, JSH19	4
JSH08	JSH 09, JSH 15	2
QSH01	JSH06, JSH12, QSH13, QSH07, QSH10, MH01, MH04, JG02, JG03, JG09, JG11, JG13, JG14	13
QSH02	QSH04, QSH08	2
QSH05	JG04	1
QSH06	QSH09	1
JG06	JG15, JG18, JG19, JG20	4
ZM03	ZM07, ZM13, ZM14, ZM20, ZM21, ZM22, ZM23, ZM24, ZM25, ZM27, ZM31, ZM40, ZM53, ZM56, ZM57, ZM59	16
